# Supplementary material for: TGFβ1 Secreted by Cancer-Associated Fibroblasts as an Inductor of Resistance to Photodynamic Therapy in Squamous Cell Carcinoma Cells
Source: Cancers (Basel). 2021 Nov 10;13(22):5613. doi: 10.3390/cancers13225613 (PMC8616019; doi:10.3390/cancers13225613)
Supplement: Supplementary file 1 [file cancers-13-05613-s001.zip › cancers-1457694-supplementary.pdf]

cancers

MDPI

Supplementary Materials: TGFβ1 Secreted by Cancer-Associated Fibroblasts as an Inductor of Resistance to Photodynamic Therapy in Squamous Cell Carcinoma Cells

María Gallego-Rentero, María Gutiérrez-Pérez, Montserrat Fernández-Guarino, Marta Mascaraque, Mikel Portillo-Esnaola, Yolanda Gilaberte, Elisa Carrasco and Ángeles Juarranz

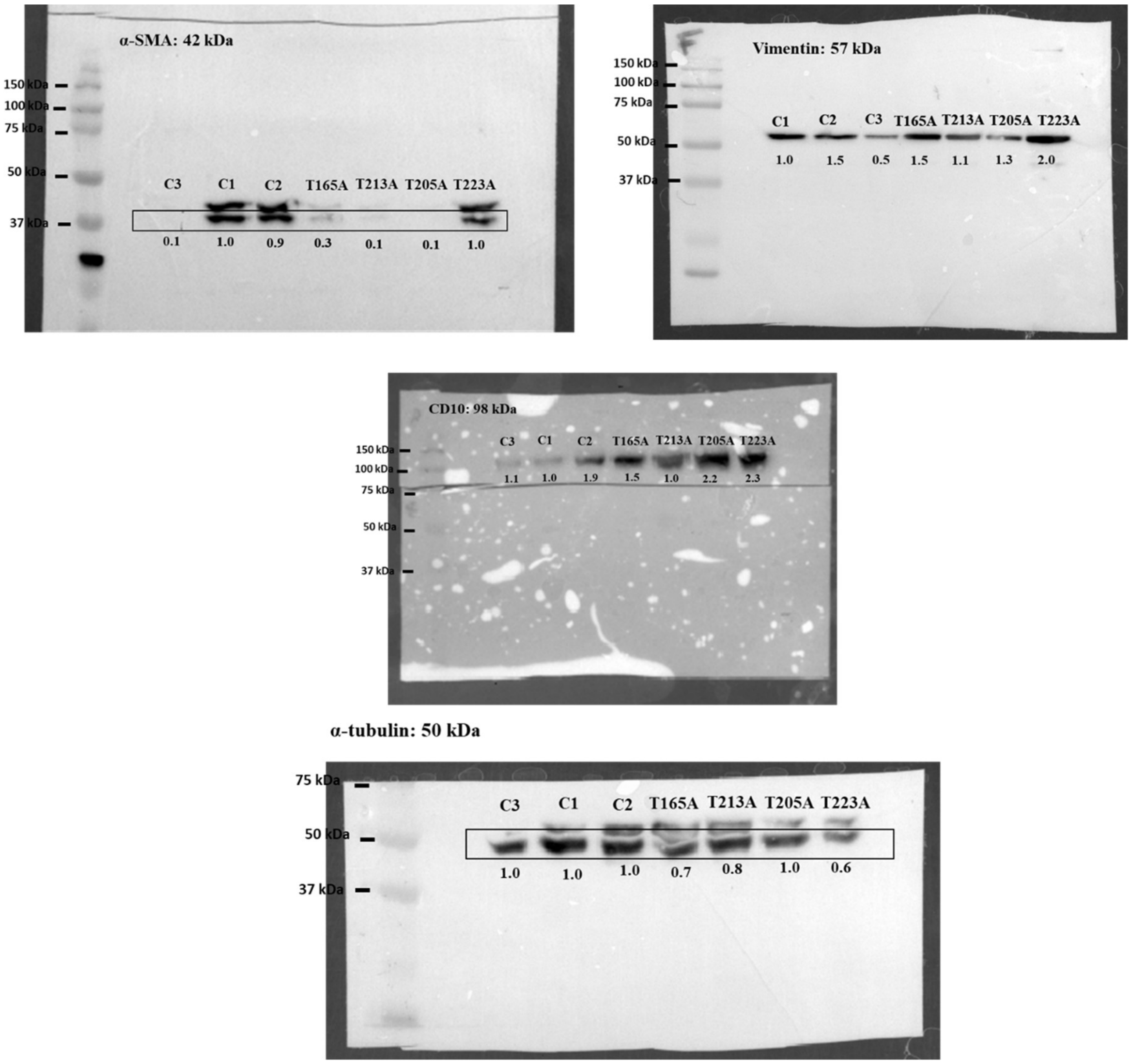

Figure S1. Whole Western blot membranes from Figure 2a.

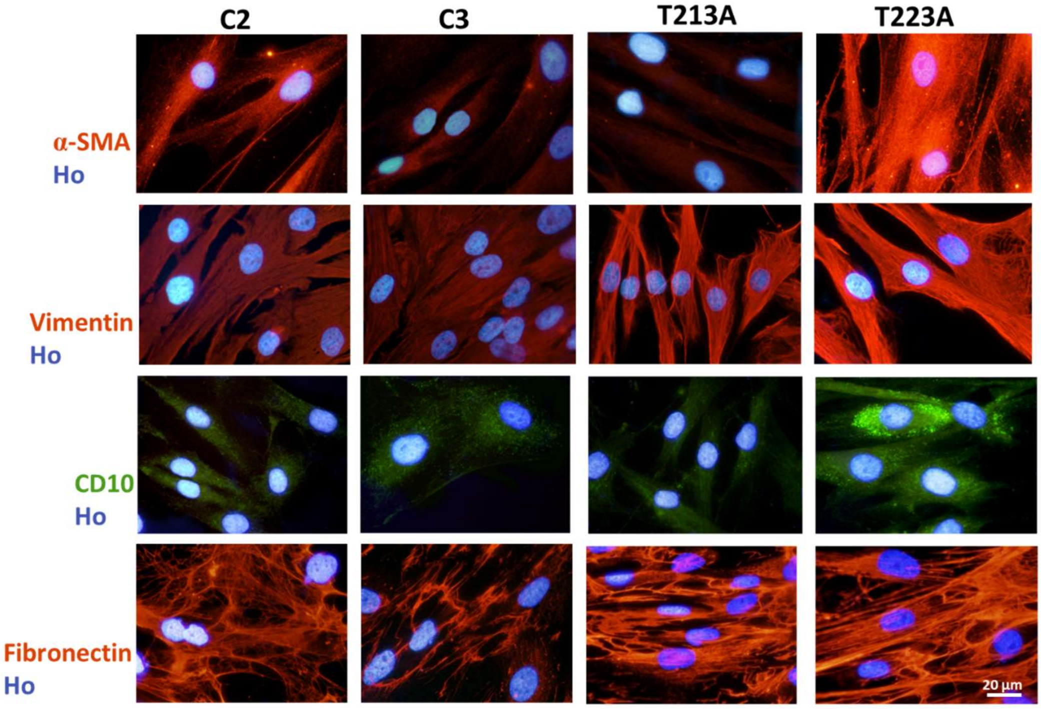

Figure S2. Analysis of the expression of different CAF markers by IF in all the control and CAF fibroblast cultures included in the study.

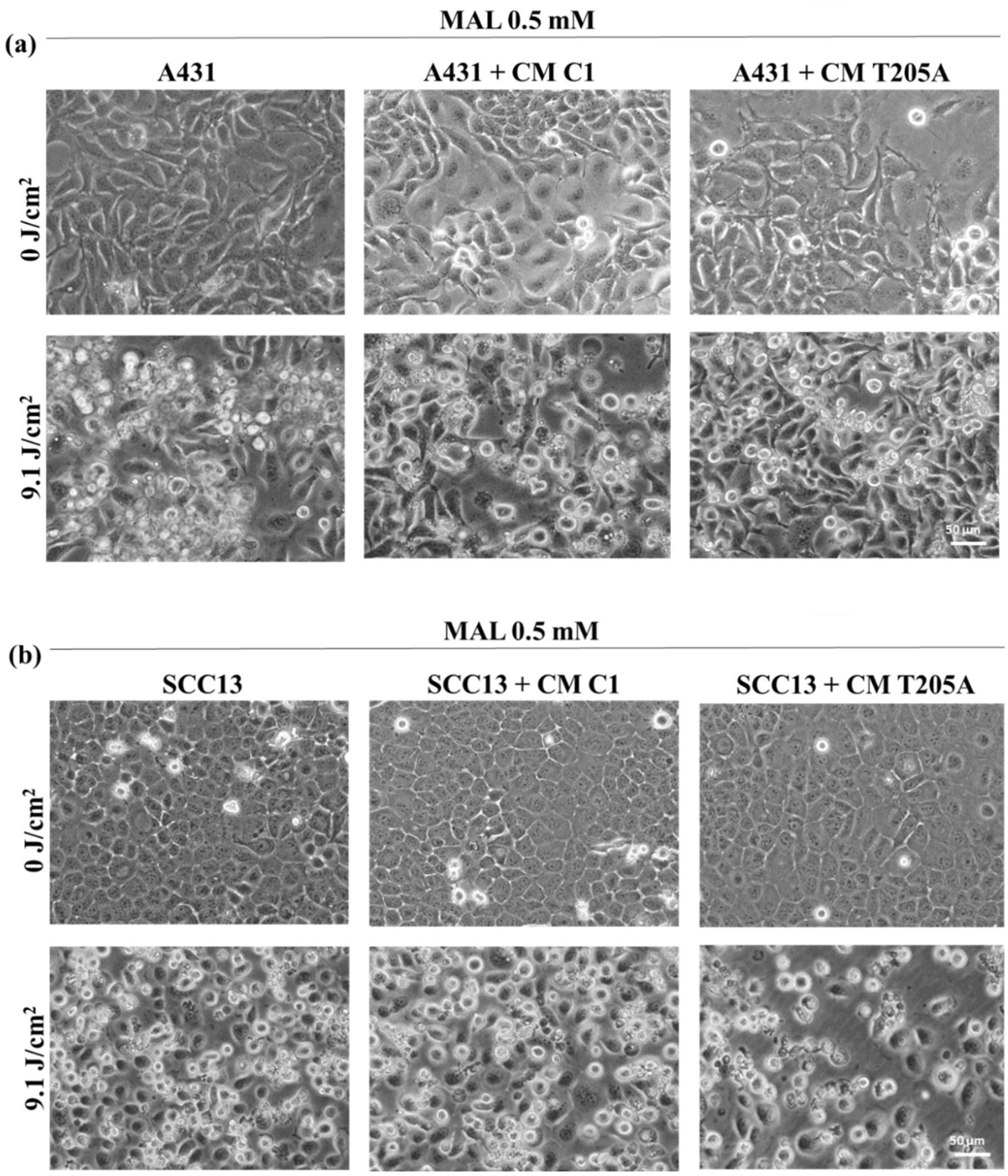

Figure S3. Phase contrast images illustrating the induction of cell death in A431 cells (a) and SCC13 cells (b) by PDT alone or in the presence of CM from control fibroblasts (C1) or from CAFs (T205A).

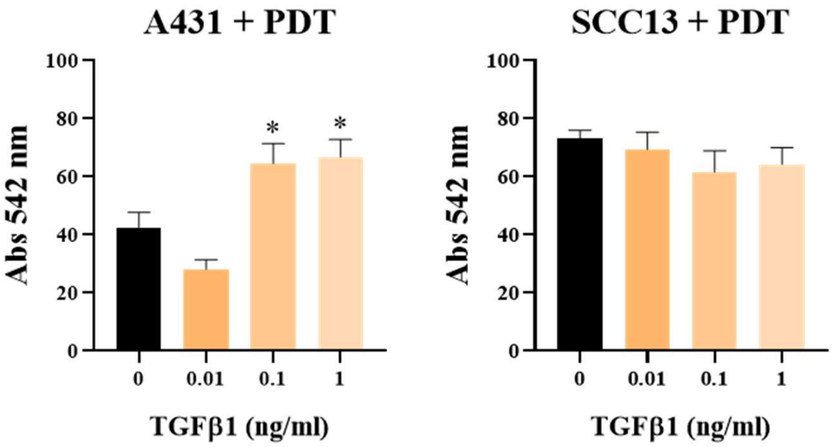

Figure S4. Effect of exogenous recombinant TGFβ1 on the resistance of cSCC cells to PDT. SCC cells were incubated for 48 h with TGFβ1 and treated with PDT (0.5 mM MAL, 6.1 J/cm2 light dose) and cell viability was assessed by MTT. TGFβ1 induced resistance only in A431 cells at 0.1 and 1 ng/ml. Error bars denote ± S.E.M. ($n = 3$, one-way ANOVA, *: $p < 0.05$).

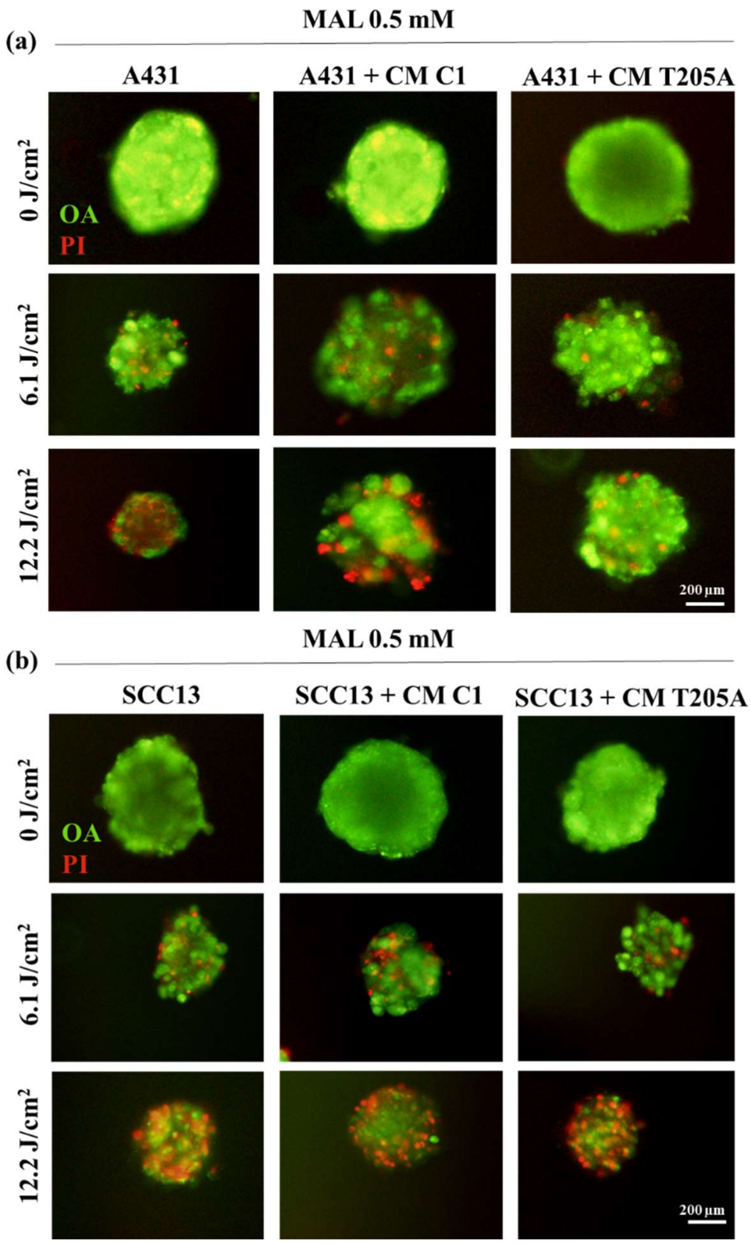

Figure S5. Cell viability in of A431 (a) and SCC13 (b) spheroids in response to different doses of PDT alone or in the presence of CM from control fibroblasts (C1) or from CAFs (T205A).

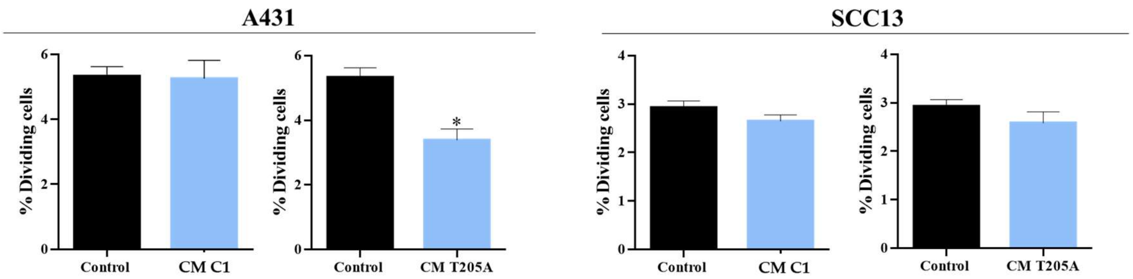

Figure S6. Comparative analysis of the mitotic index in A431 and SCC13 cells under standard culture conditions or in the response to the incubation with CM from control fibroblasts (C1) or from CAFs (T205A).

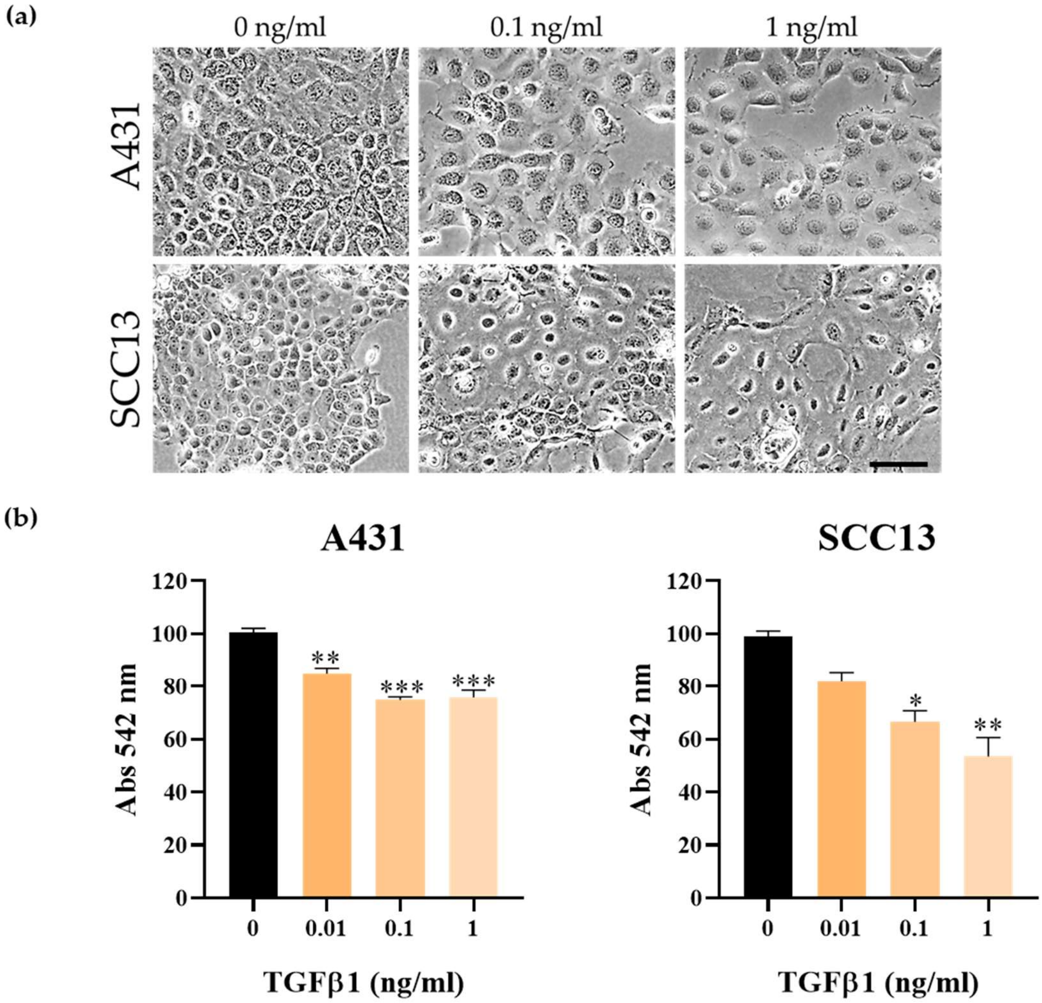

Figure S7. Effect of exogenous TGFβ1 on cSCC cell lines. The cell cultures were incubated for 48 h with recombinant TGFβ1. (a) A shift into more flattened morphologies was observed by phase contrast microscopy in cells treated with TGFβ1. Importantly, the frequency of cell death was not found increased compared to untreated cells. Scale bar = 100 μm. (b) The MTT assay revealed significantly reduced absorbance in the cultures exposed to TGFβ1. Error bars denote ± S.E.M. (n = 3, one-way ANOVA, *: $p < 0.05$; **: $p < 0.01$; ***: $p < 0.001$).

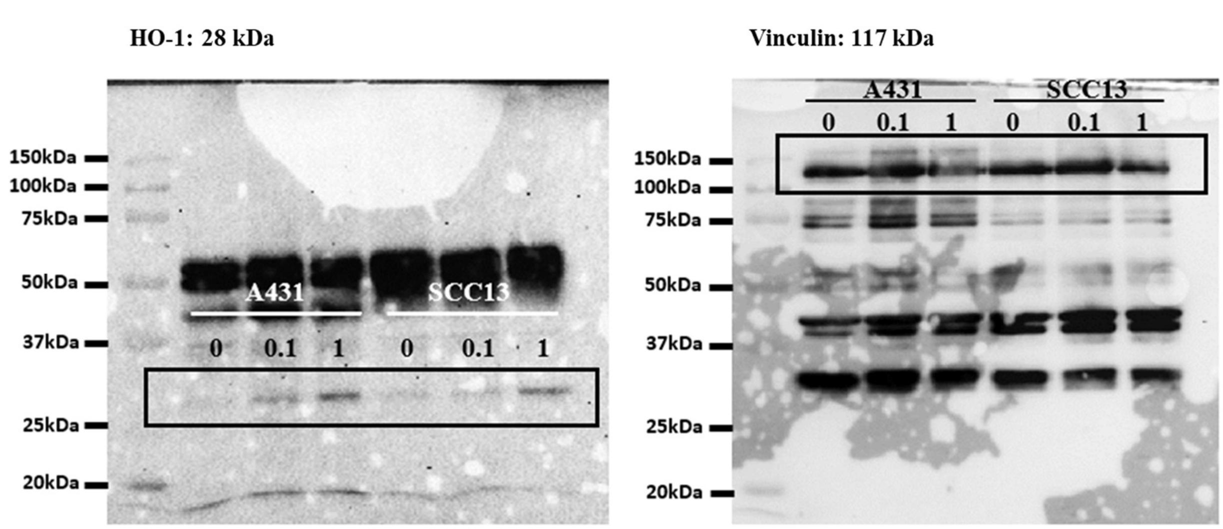

Figure S8. Whole Western blot membranes from Figure 8b.
